# Use of Rituximab After Orbital Decompression Surgery in Two Grave’s Ophthalmopathy Patients Progressing to Optic Neuropathy

**DOI:** 10.3389/fendo.2020.583565

**Published:** 2020-10-26

**Authors:** Benping Zhang, Yaling Li, Weijie Xu, Bei Peng, Gang Yuan

**Affiliations:** ^1^Department of Endocrinology, Tongji Hospital, Tongji Medical College, Huazhong University of Science and Technology, Wuhan, China; ^2^Department of Critical Care Medicine, Wuhan No.1 Hospital, Wuhan, China; ^3^Department of Endocrinology, Taikang Tongji (Wuhan) Hospital, Wuhan, China

**Keywords:** Grave’s disease, Grave’s ophthalmopathy, Dysthyroid optic neuropathy, orbital decompression, rituximab

## Abstract

**Background:**

While orbital decompression can alleviate optic nerve compression and prevent further vision loss in dysthyroid optic neuropathy (DON), it cannot relieve inflammatory symptoms. Very high doses of intravenous glucocorticoids (GCs) are the first-line therapy for DON; however, the effective rate is only 40% and might be much lower in patients who fail high-dose GC pulse therapy and progressed to DON. The results of two case series studies indicated that rituximab treatment had a much better curative effect compared to very high doses of intravenous GCs, but some patients required urgent orbital decompression after rituximab injection because rituximab might lead to the release of cytokines, aggravated intraorbital edema, and further vision loss.

**Methods:**

We retrospectively studied the therapeutic process of two Grave’s ophthalmopathy (GO) patients complicated with DON who failed high-dose GC pulse therapy and underwent orbital decompression. Both patients received single-dose (500 mg) rituximab treatment.

**Results:**

During more than 2 years of follow-up, rituximab treatment exhibited significant improvement in inflammatory symptoms, as manifested by a substantial decrease in Clinical Activity Score (CAS); meanwhile, the vision of both patients improved significantly and their diplopia was relieved.

**Conclusions:**

The results of this study were consistent with those of two previous case series studies indicating the significant and lasting effect of rituximab treatment on DON, especially for patients with GC resistance or recurrence after GC therapy. Orbital decompression before rituximab treatment might reduce the incidence of rapid vision loss and urgent orbital decompression surgery caused by aggravated orbital edema after rituximab injection; however, the necessity for preventive decompression surgery requires further study.

## Introduction

Grave’s disease (GD) is an autoimmune disease involving the thyroid, skin, and orbit, with an incidence in the adult population of 1–2% (1). Grave’s ophthalmopathy (GO) is the most frequent extrathyroidal manifestation of GD, occurring in up to 50% of GD patients throughout the course of the disease (2). GO is generally self-limiting, with the signs and symptoms improving naturally or following thyrotoxicosis control, smoking cessation, and local treatment (3). However, for some patients, the signs and symptoms persist or aggravate gradually, which require specific treatment besides smoking cessation and anti-thyroid drug administration (4).

The pathogenesis of GO is incompletely understood, although immunological cross-reactivity between the thyroid and orbital antigens may play a key role, and disorders of inflammatory cytokines, thyrotropin receptor autoantibodies, and immunoglobulins targeting the insulin-like growth factor 1 receptor may be correlated with GO (2, 5). Hence, the current internal medicine treatments for GO mostly target immunological disorders, especially for moderate-to-severe and sight-threatening GO.

High-dose intravenous glucocorticoid (GC) pulse treatment is the first-line treatment for moderate-to-severe and active GO, with a 4.5 g cumulative dose of methylprednisolone divided into 12 weekly intravenous injections recommended. A higher dose is acceptable for severe forms; however, the cumulative doses should not exceed 8.0 g (4, 6). The response rate of high-dose intravenous GCs pulse treatment is 70–80% (4, 6, 7); however, in clinical practice, many patients responding well to intravenous GCs may re-develop active disease after therapy completion. For patients with a recurrence or poor response to intravenous GCs, shared decision-making with patients to select an appropriate second-line treatment is recommended (6). A second course of intravenous GCs, orbital radiotherapy, combination use of cyclosporine and oral GCs, and rituximab are the most common second-line treatments (6).

As an alternative second-line treatment for GO, rituximab has been proven to be effective and safe in patients with GO who fail high-dose intravenous GC pulse treatment and shows potential to become a first-line treatment (6). For GO patients progressing to DON, non-control studies have also shown that rituximab was effective in relieving DON; however, a small number of patients in the studies required urgent orbital decompression surgery after receiving rituximab injection as rituximab may have aggravated intraorbital edema (8–10). This finding is consistent with the recommendation in the 2016 European Group of Graves’ Orbitopathy Guidelines for the Management of GO that rituximab should not be used in patients with impending DON (6). Thus, the use of rituximab in patients with GO complicated with DON requires further clinical studies.

This study retrospectively analyzed the therapeutic processes of two GO patients who failed high-dose intravenous GC pulse treatment and progressed to DON. Although a very high dose of intravenous GCs is the first-line treatment for DON, patient 1 was not sensitive to GCs and the cumulative doses of GCs exceeded 8 g. Two patients underwent orbital decompression surgery first to avoid further vision loss. However, there was no significant improvement, especially the clinical activity score (CAS) and diplopia. Moreover, the recovery of vision was not ideal. Thus, rituximab treatment was initiated. During 2 years of follow-up after rituximab treatment, both patients achieved stable and significant remission.

## Case Descriptions

Both patients were initially diagnosed with moderate-to-severe and active GO and received high-dose intravenous GC pulse treatment. The patients also received basic treatments including selenium supplementation and artificial tears. Orbital decompression surgery and sequential rituximab were initiated when the patients suffered from DON and resisted to GCs or the total methylprednisolone dose exceeded 8g. All patients received a single dose of 500 mg rituximab as a slow intravenous infusion, with 5 mg of dexamethasone to prevent allergic reactions.

Case 1: A 54-year-old man (non-smoker) was diagnosed with GD and GO in December 2017 at a local hospital, where he received methimazole treatment only. Three months later, he was transferred to Tongji Hospital, Wuhan, China, for further treatment due to rapidly declining vision (OD 0.3, OS 0.08), eyeball movement disorder, and inability to close his eyelid. The patient was then diagnosed with DON, with a CAS of 7/7. Two days after transfer to this hospital, we initiated high-dose intravenous GC pulse treatment (0.5 g methylprednisolone seven times) and performed bilateral orbital decompression and eyelid margin suture surgery as ophthalmologic examination showed persisting DON with no significant improvement in vision. The orbital decompression surgery was balanced decompression of the inner and outer orbital walls combined with lipectomy, part of the bone in both medial and lateral orbital walls and about 3ml of adipose tissue were removed in the surgery. After the above treatment, the patient’s CAS decreased to 5/7, and ophthalmologic examination showed normal intraocular tension; however, the eyeball movement disorder remained and his vision did not improve (OD 0.1, OS 0.1). The patient then left the hospital and received oral GC treatment at home (methylprednisolone 40 mg/d for 1 week and 36 mg/d for another week). Two weeks later, the patient returned to the hospital. Ophthalmologic examination showed a CAS of 6/7; therefore, we administered an eighth intravenous GC treatment (0.5 g methylprednisolone). His vision improved 7 days after the intravenous GC treatment (OD 0.5, OS 0.6), but the CAS was still 6/7, and the eyeball movement disorder and inability to close the eyelids did not improve. Finally, we decided to administer rituximab treatment (a single dose of 500 mg) and oral GC treatment simultaneously.

Case 2: A 58-year-old man (smoker, quit smoking 10 years ago) was diagnosed with hyperthyroidism and moderate-to-severe GO in March 2017 at the local hospital, where he received methimazole and intravenous GC treatment (11 weekly intravenous injections of 0.5 g methylprednisolone), without significant improvement. The patient was then transferred to Tongji Hospital, Wuhan, China. Ophthalmologic examination showed eyeball movement disorder; both eyes had 1.0 vision, and his CAS was 5/7. We administered intravenous GC treatment (12 weekly intravenous injections of 0.25 g methylprednisolone) combined with right orbital radiotherapy (2 Gy daily for 10 days). After the above treatment, his vision was normal (OD 1.2, OS 1.2), the intraocular tension was also normal, and the CAS decreased to 2/7. However, the disease relapsed approximately 2 months after completion of intravenous GC therapy, which manifested as severe exophthalmos, diplopia, vision decrease (OD 0.1, OS 1.0), and inability to close his eyelid. The CAS increased to 7/7. The patient was then diagnosed with DON and right orbital decompression surgery was performed. The surgical type and methods were similar to those described for patient 1. After orbital decompression surgery, his vision improved slightly (OD 0.4, OS 1.0); however, the CAS and diplopia did not improve. Therefore, we decided to administer rituximab treatment (a single dose of 500 mg) and oral GC treatment.

**Figure 1:**
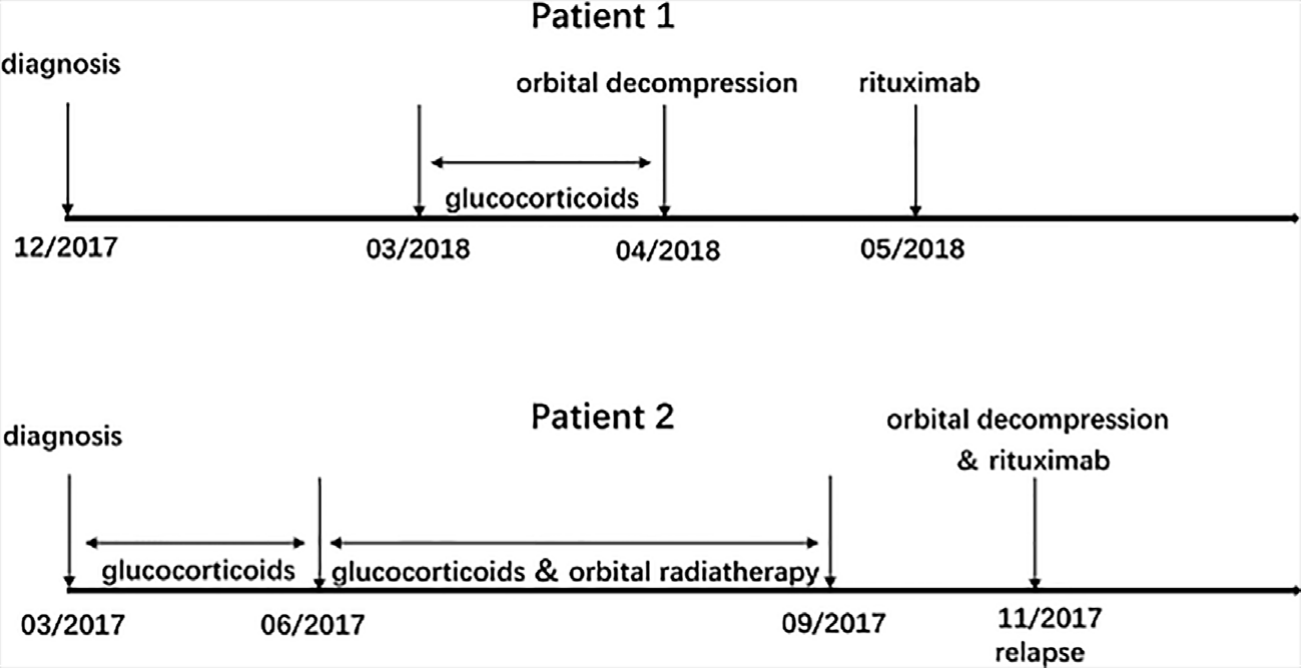
Treatment process: corticosteroids, orbital decompression, and rituximab for patient 1 and patient 2.

The treatment process of patient 1 and patient 2 was summarized in **Figure 1**. **Table 1** showed the baseline data of patients before rituximab treatment, **Table 2** showed in detail the patients undergoing orbital decompression surgery and rituximab treatment, and **Table 3** showed the changes in patients' vision before rituximab treatment.

**Table 1:** Description of the two patients treated with Rituximab.

Patient number	Patient 1	Patient 2
Age (year)	54	58
Gender	Male	Male
Smoking	No	Yes
Quit smoking	N/A	10 years ago
Endocrinological treatment of GD	Methimazole	Methimazole
Chronology of GO in relation to GD	With GD	With GD
Corticosteroids therapy (time relative to GO diagnosis)	3 months	0 month
Corticosteroids therapy (duration time)	8 weeks	23 weeks
Total dose of intravenous corticosteroids dose (methylprednisolone, MPS)	4.0g	8.5g
Other immunosuppressive treatments before RTX	Oral MPS for 2 weeks, total dose 532mg	None
Orbital radiotherapy	None	2Gy/time × 10 times
Additional treatments	Selenium supplements	Selenium supplements

GD, Grave’s disease; GO, Grave’s ophthalmopathy; RTX, rituximab; MPS, methylprednisolone; N/A, not available.

**Table 2:** Orbital decompression and Rituximab treatment details of the two patients.

Patient number	Patient 1	Patient 2
Reason for orbital decompression	DON, no improvement after intravenous MPS	Relapse and DON developed after intravenous MPS and orbital radiotherapy
Total dose of MPS before orbital decompression	3.5g	8.5g
Duration of MPS use before orbital decompression	7 weeks	23 weeks
Orbital decompression (time relative to DON diagnosis)	At 7 weeks	At 1 week
Immunosuppressive treatments after orbital decompression	Intravenous MPS 0.5g and oral MPS	None (prior to RTX)
RTX therapy (time related to orbital decompression)	At 52 days	At 4 days
RTX therapy (time related to diagnosisi)	5 months	8 months
Reason for RTX therapy	Failure of glucocorticoids treatment	MPS dose exceeded 8g
RTX dose	500mg only once	500mg only once
Immunosuppressive treatments after RTX	Oral MPS	Oral MPS

DON, dysthyroid optic neuropathy; MPS, methylprednisolone; RTX, rituximab.

**Table 3:** Summary of changes in vision before RTX treatment.

	Vision	
	Right	Left
Patient 1		
Before high-dose GCs pulse treatment	0.3	0.08
After 7 times high-dose GCs pulse treatment	0.1	0.1
After bilateral orbital decompression and the eighth high-dose GCs pulse treatment (before RTX)	0.5	0.6
Patient 2		
Before high-dose GCs pulse treatment combined with right orbital radiotherapy	1.0	1.0
After high-dose GCs pulse treatment combined with right orbital radiotherapy	1.2	1.2
Disease relapse	0.1	1.0
After right orbital decompression (before RTX)	0.4	1.0

RTX, rituximab; GCs, glucocorticoids.

## Results

**Figure 2:**
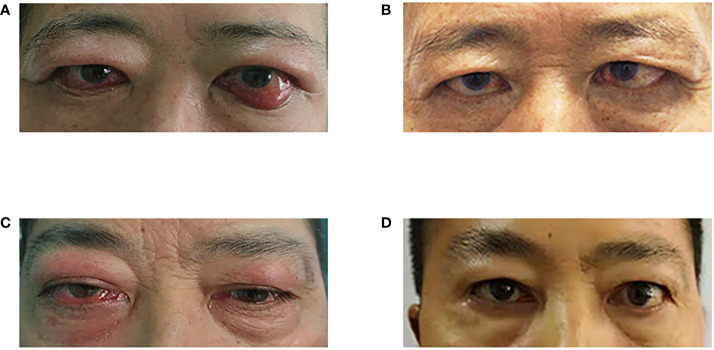
Changes in the appearance of the two patients' eyes. **(A)** Before rituximab treatment (patient 1); **(B)** 24 months after rituximab treatment; **(C)** Before rituximab treatment (patient 2); **(D)** 26 months after rituximab treatment (patient 2).

As shown in **Table 4** and **Figure 2**, rituximab treatment was administered after orbital decompression and showed significant improvement in inflammatory symptoms, as manifested by a substantial decrease in CAS. Meanwhile, the vision of both patients improved significantly, and their diplopia was relieved after rituximab treatment.

**Table 4:** Results of the therapy with rituximab.

	CAS score (/7)		Vision		IPO (mmHg)		Proptosis (mm)		Diplopia
	Right	Left	Right	Left	Right	Left	Right	Left	
Patient 1									
Before RTX	5	6	0.5	0.6	19	15	21	20	Yes
5 months after RTX	2	3	1.0	0.8	18	14	20	21	Alleviated
Last examination 24 months after RTX	2	2	1.0	0.8	18	15	19	20	Alleviated
Patient 2									
Before RTX	7	3	0.4	1.0	21	19	19	19	Yes
6 months after RTX	2	0	0.8	1.0	19	17	18	18	Alleviated
Last examination 26 months after RTX	0	0	1.0	1.2	19	17	18	19	No

CAS, Clinical Activity Score; IPO, intraocular pressure; RTX, rituximab.

In patient 1, the CAS decreased from 5/7 to 2/7 (OD) and from 6/7 to 3/7 (OS) 5 months after rituximab treatment, with a final CAS of both eyes of 2/7 at the last follow-up to 24 months after rituximab treatment. His vision had improved from 0.5 to 1.0 OD and from 0.6 to 0.8 (OS) at the last follow-up. The severity of diplopia decreased but persisted until the last follow-up. Slight improvements in intraocular pressure and proptosis were also observed.

In patient 2, similar to patient 1, inflammatory signs and vision improved significantly after rituximab treatment, while intraocular pressure and proptosis improved slightly. Specifically, the CAS decreased from 7/7 to 2/7 (OD) 6 months after rituximab treatment, with a final CAS of 0/7 (OD) at the last follow-up. The patient’s vision had improved from 0.4 to 1.0 OD and from 1.0 to 1.2 (OS) at 26 months after rituximab treatment. In addition, diplopia was alleviated at 6 months and completely relieved at 26 months after rituximab treatment.

## Discussion

Rituximab is a mouse anti-human monoclonal antibody that directly targets CD20, a B-lymphocyte-specific antigen (11). It was initially developed and used in patients with CD20-expressing lymphoid malignancies, including chronic lymphocytic leukemia and B-cell non-Hodgkin lymphoma. Rituximab induces cell apoptosis by binding to the CD20 membrane antigen in both normal and malignant B cells (12). In addition, it significantly depletes lymphocytes not only in the blood but also in the target tissue, and the effect remained significant 2 months after rituximab infusion (13). Based on the pharmacological mechanism of rituximab, clinical trials on autoimmune diseases have indicated the potential use of rituximab as a candidate treatment for autoimmune diseases (14).

In 2006, two case reports indicated that corticosteroid-resistant GO might be significantly relieved after treatment with rituximab (15, 16). Following the two successful treatment cases, several non-controlled studies on the effects of rituximab in GO have been published, with most showing that rituximab can be used in active moderate-to-severe GO, especially when intravenous methylprednisolone therapy fails (5). In one study, Salvi et al. reported good therapeutic effects after rituximab treatment and improved activity and severity of GO in 98 and 91% of 43 GO patients who failed to respond to corticosteroids, respectively (17). In recent years, the results of two randomized clinical trials of rituximab as a first-line treatment in moderate-to-severe and active GO were published. In one study, Salvi et al. reported that GO was more significantly inactivated after 500 or 2,000 mg (administered twice over 2 weeks) rituximab injection (100 *vs.* 69% after 7.5 g intravenous methylprednisolone) at 24 weeks (18). However, another study by Stan et al. did not observe differences in either short- or long-term (24- or 52-week) outcomes between rituximab and placebo; meanwhile, two patients developed DON after rituximab. One possible explanation for the occurrence of DON is that the release of cytokines induced by rituximab treatment resulted in aggravated intraorbital edema, which transformed subclinical DON to DON (5, 19). The contradictory results of the two clinical trials suggest the need for more evidence before rituximab can be implemented as a first-line treatment for GO. However, the 2016 European Group of Graves’ Orbitopathy Guidelines for the Management of Graves’ orbitopathy recommended that, although it is still difficult to say which second-line option is more effective because of the limited evidence regarding differences in efficiency difference, treatment has relatively more evidence suggesting that rituximab is a good option for active moderate-to-severe GO when high-dose intravenous GC pulse treatment fails (6).

In GO patients progressing to DON, very high doses of intravenous GCs (*e.g.* 500–1,000 mg of methylprednisolone for 3 consecutive days) is the first-line therapy; however, the effective rate is only 40%, and many patients require urgent decompression surgery when the response is poor (6). In the formulation of the patients’ treatment plans in the present report, we considered that very high doses of intravenous GCs might be ineffective in patient 1 as he showed resistance GCs, which manifested as non-remission of the disease after seven intravenous GC treatments. Meanwhile, very high doses of intravenous GCs might have led to serious side effects in patient 2 as the cumulative doses of GCs exceeded 8 g. To relieve compression of the optic nerve and avoid further loss of vision, we performed orbital decompression surgery first instead of administering very high doses of intravenous GCs. However, as it was predictable before the surgery that the patients’ condition, especially the inflammatory symptoms and eye movement disorder, did not significantly improve as orbital decompression could only relieve orbital pressure and optic nerve compression; thus, further drug treatment was urgently required.

No evidence-based medical recommendations exist regarding the further treatment of GO patients complicated with DON who fail high-dose GC pulse therapy and require orbital decompression. We finally decided to administer rituximab based on the results of several non-control studies that indicated a better curative effect for rituximab treatment than for first-line therapy in GO patients with DON (8–10). Among these studies, Chong et al. successfully treated four patients who failed to respond to GCs and developed DON. In these patients, the CAS decreased significantly 2 months after rituximab treatment and remained so in all patients. The vision also improved bilaterally in all four patients. One of the four patients received decompression surgery and fractionated orbital irradiation 2 months before rituximab infusion, and another patient received urgent decompression surgery 12 days after the first rituximab infusion due to continued DON (8). Two other studies by Salvi et al. and Mitchell et al. also reported the successful treatment of five patients with DON, with significantly improved disease activity and vision in all five patients, similar to Chong’s study. One of the five patients received urgent decompression surgery after the first rituximab infusion (9, 10). The longest interval between rituximab infusion and orbital decompression surgery was 2 months. Gess et al. administered rituximab to a patient with GO with DON who had failed very high-dose intravenous GCs. The patient improved initially but subsequently worsened 2 months later and underwent orbital decompression surgery (13).

The common characteristics of both patients in this study were significant enlarged extraocular muscle and orbital fat, which led to optic nerve compression and progression of DON. Previous studies revealed that the enlargement of extraocular muscle and orbital fat was associated with hyaluronic acid (HA) accumulation in the muscles and connective tissues and adipogenesis (20, 21). B cells play an important role in HA accumulation and adipogenesis. Briefly, B cells can present self-antigen and activate T cells through the complement receptor 2 (CR2, CD35), then activate helper T cells, recognize TSHR, ligate TSHR and TRAb together, and enhance hyaluronic acid (HA) production and adipogenesis (21, 22). Therefore, rituximab inhibits the activation of T cells by B-cell depletion and blockade of antigen presentation, which may be the main reason why rituximab is more effective compared with GC.

In conclusion, the treatment processes of these two patients indicated that rituximab might be safe and persistently effective for GO patients complicated with DON who fail high-dose GC pulse therapy and require orbital decompression. This finding is consistent with those of two previous non-control studies suggesting that rituximab treatment may have a better curative effect than very high doses of intravenous GCs. However, rituximab treatment may lead to the release of cytokines, aggravated intraorbital edema, and rapid vision loss. Orbital decompression before rituximab injection might mitigate this side effect of rituximab; thus, the patients in this study might have obtained additional benefits from orbital decompression, although the surgeries were not performed preventively. The necessity for prophylactic orbital decompression surgery before rituximab injection requires additional evidence.

## Data Availability Statement

The raw data supporting the conclusions of this article will be made available by the authors, without undue reservation.

## Ethics Statement

The studies involving human participants were reviewed and approved by Ethics Commission of Tongji Hospital, Tongji Medical College, Huazhong University of Science and Technology. The patients/participants provided their written informed consent to participate in this study. Written informed consent was obtained from the individual(s) for the publication of any potentially identifiable images or data included in this article.

## Author Contributions

BZ: Data curation, Project administration, Formal analysis, Writing—original draft, Writing—review and editing. YL: Data curation, Writing—review and editing. WX: Data curation, Writing—review and editing. BP: Data curation, Writing—review and editing. GY: Conceptualization, Data curation, Project administration, Formal analysis, Investigation, Methodology, Project administration, Validation, Writing—review and editing.

## Conflict of Interest

The authors declare that the research was conducted in the absence of any commercial or financial relationships that could be construed as a potential conflict of interest.

## Abbreviations

CAS, Clinical Activity Score; DON, dysthyroid optic neuropathy; GC, glucocorticoid; GD, Grave’s disease; GO, Grave’s ophthalmopathy.
